# GATA1 and PU.1 Bind to Ribosomal Protein Genes in Erythroid Cells: Implications for Ribosomopathies

**DOI:** 10.1371/journal.pone.0140077

**Published:** 2015-10-08

**Authors:** Elsa P. Amanatiadou, Giorgio L. Papadopoulos, John Strouboulis, Ioannis S. Vizirianakis

**Affiliations:** 1 Laboratory of Pharmacology, Department of Pharmaceutical Sciences, Aristotle University of Thessaloniki, Thessaloniki, Greece; 2 Division of Molecular Oncology, Biomedical Sciences Research Center "Alexander Fleming", Vari, Greece; University of Thessaly, Faculty of Medicine, GREECE

## Abstract

The clear connection between ribosome biogenesis dysfunction and specific hematopoiesis-related disorders prompted us to examine the role of critical lineage-specific transcription factors in the transcriptional regulation of ribosomal protein (RP) genes during terminal erythroid differentiation. By applying EMSA and ChIP methodologies in mouse erythroleukemia cells we show that GATA1 and PU.1 bind in vitro and in vivo the proximal promoter region of the RPS19 gene which is frequently mutated in Diamond-Blackfan Anemia. Moreover, ChIPseq data analysis also demonstrates that several RP genes are enriched as potential GATA1 and PU.1 gene targets in mouse and human erythroid cells, with GATA1 binding showing an association with higher ribosomal protein gene expression levels during terminal erythroid differentiation in human and mouse. Our results suggest that RP gene expression and hence balanced ribosome biosynthesis may be specifically and selectively regulated by lineage specific transcription factors during hematopoiesis, a finding which may be clinically relevant to ribosomopathies.

## Introduction

Ribosome biogenesis is a highly coordinated process leading to the stoichiometric assembly of all ribosomal components. In eukaryotes, 4 rRNAs and 80 different ribosomal proteins (RPs) are produced, processed and assembled into functional ribosomes [1, 2]. RP biosynthesis is regulated at multiple levels by transcriptional, translational and post translational mechanisms so that RP balance is achieved [3, 4]. In higher eukaryotes little is known about the transcriptional regulation of RP genes which are scattered in different chromosomes and possess distinct promoters sharing certain structural features but no common motifs [5, 6].

Despite ubiquitous RP gene expression and functions across all tissues, RP gene haploinsufficiency leading to perturbation of balanced ribosome assembly results in clinical syndromes with highly specific phenotypes in man, including bone marrow aplasia and cancer susceptibility [7]. For example, RPS14 haploinsufficiency leads to hypoplastic/macrocytic anemia in 5q deletion (5q^-^) syndrome, an acquired hematological disorder [8, 9]. In addition, Diamond-Blackfan Anemia (DBA) is a genetic syndrome caused by heterozygous mutations in several RP genes involved in the biogenesis of the small and large ribosomal subunits, such as RPS10, RPS26, RPS24, RPS17, RPS7, RPL35a, RPL11, RPL5, RPL26, RPL15 and RPS19, which account for ~60–70% of DBA cases [10–12]. DBA is predominantly characterized by anemia, macrocytosis and reticulocytopenia, however its molecular pathogenesis pathways remain poorly understood [13–15]. It is known that DBA specifically relates to the decline or absence of erythroid progenitors in an otherwise normocellular bone marrow, with the defect shown to occur at the stage of BFU-E and early CFU-E progenitors failing to differentiate to mature red blood cells [16, 17].

A number of non-mutually exclusive mechanisms have been proposed to account for the hematopoietic specificity of DBA, including an increased sensitivity of erythroid precursors to apoptosis and the high demands imposed on protein synthesis by hemoglobin accumulation [7, 18, 19]. Indeed, ribosome number and activity appear to be heavily modulated during physiological terminal erythroid differentiation, in that ribosome numbers peak in early proerythroblast differentiation, followed by a gradual decline in RP gene transcription and ribosome formation with terminal differentiation [20, 21]. Thus, given the dynamic nature of ribosome number and function during erythroid maturation and the specific hematopoietic phenotypes in RP gene haploinsufficiency, we reasoned that hematopoietic transcription factors (TFs) are implicated in balanced ribosome biosynthesis during erythropoiesis by specifically regulating RP gene transcription. This is supported by the recent identification of rare GATA1 mutations, resulting in the expression of a short isoform of the GATA1 protein (GATA1s) in DBA patients with no detectable mutations in RP genes [22, 23]. Despite this evidence, an investigation of potential RP gene regulation in erythroid cells by hematopoietic TFs has not been systematically undertaken in the past.

Here, we describe the binding of several RP genes, including genes mutated in DBA, by the GATA1 and PU.1 TFs in murine erythroleukemic (MEL) cells, a well characterized cellular model of erythropoiesis. GATA1 and PU.1 are considered master regulators of the erythroid and myeloid-lymphoid lineage transcription programs, respectively, and are known to be functionally cross-antagonistic [24]. We also used publicly available ChIPseq data to determine GATA1 and PU.1 occupancies in all RP gene promoter regions in mouse and human erythropoiesis models of fetal and adult origin. We also related GATA1 occupancy profiles to RP gene expression levels during late erythroid differentiation in mouse and human. Our results support the notion that GATA1 and PU.1 are implicated in the transcriptional regulation of RPs in hematopoiesis and shed new light on the potential molecular links between ribosome production and erythropoiesis.

## Materials and Methods

### Cell culture

Mouse erythroleukemic (MEL) cells (clone 745-PC–4) were derived from the MEL 745 cell line, originally isolated by Friend et al. [25] and were maintained and induced to differentiate with 5mM of HMBA (N,N-Hexamethylene-bis- acetamide, Sigma) as previously described [26]. Cell culture density and differentiation (hemoglobin production) were assessed as previously described [26].

### Protein extract preparation

Total MEL cell extracts were prepared by lysis with RIPA buffer with protease inhibitors, followed by sonication of 5cycles/20seconds each and collection of the supernatant by centrifugation. Nuclear extracts were prepared using the Dignam method [27]. Protein concentration was determined using the Bradford assay.

### Western blot analysis

Western blot analysis was performed using 10–20μg of protein/sample, as previously described [28]. Primary antibodies included anti-GATA1 (sc–265), anti-PU.1 (sc–352), anti-RPS19 (sc–134779), anti-tubulin α (sc–51503), all purchased from Santa Cruz Biotechnology. Anti-nucleophosmin was a gift by Professor Pui-Kwong Chan, Baylor College of Medicine, TX.

### Electrophoretic mobility shift assay (EMSA)

EMSA reactions using 10μg of MEL nuclear extracts were carried out as previously indicated [29]. Supershift assays were done using anti GATA–1 and anti-PU.1 antibodies (see above). Competition assays were carried out using an excess of cold-competitor oligonucleotides. EMSA probes are shown in S1 Table.

### Chromatin Immunoprecipitation

ChIP assays were essentially performed as previously described [28] using chromatin from 1x10^7^ MEL cells and 4μg each of anti-GATA1 (ab11852, Abcam) and anti-PU.1 (sc–352, Santa Cruz Biotechnology). ChIP assays were carried out in biological replicates.

### Real time PCR (qPCR)

Real time PCR was carried out in triplicate with ChIPed DNA and 400nM specific primers using a SYBR Green kit (Kappa Biosystems). ChIP primer sequences are shown in S2 Table. C_T_ values for all samples were normalized to GAPDH and absolute enrichments were calculated versus input, as previously described [30]. Gene expression analysis by qRT-PCR was done with 200ng of starting RNA, reverse-transcribed (Qiagen) amplified using gene-specific primers and GAPDH for normalization (S3 Table). Standard deviation was calculated using technical triplicates and biological replicates.

### RPS19 promoter sequencing

The RPS19 proximal promoter region was amplified form MEL genomic DNA using primers Forward: 5’- AGGTGGTGGTGGCCACATGTCAT–3’ and Reverse: 5’- GTGCTCGCGAGAGCGGCTAAA -3’ (-959bp to -559bp relative to the ATG). The amplified product was sequenced (accession # LN651201) and scanned for GATA1 and PU.1 binding sites using bioinformatics tools available at http://snpper.chip.org/mapper, http://labmom.com/link/alibaba_2_1_tf_binding_prediction.

### Analysis of publicly available NGS data

GATA1 genome wide occupancies in mouse fetal liver derived erythroid cells have been previously published by our group [31]. All other ChIPseq and NGS datasets were publicly available and are listed in S4 Table. Reads alignment was performed using the bowtie.2 algorithm [32] selecting uniquely mapped reads. The mm9 and hg19 genome assemblies were used for mouse and human data, respectively. Mouse GATA1 and PU.1 target genes were extracted from S2 Table of Papadopoulos et al.[31], including total gene scores (TGS) for all genes expressed in mouse fetal liver cells. TGS of mouse datasets is calculated as the sum of ChIPseq reads mapping within a 10kb window around a gene’s transcription start site (TSS)[31]. TGS of human datasets (GATA1 in fetal and adult derived erythroblasts) is calculated as the sum of peaks called by MACS [33] within a 10kb window around the gene's TSS. Target genes are defined as having a mean normalized TGS greater than 1 (above the dataset mean) within that particular dataset. Gene expression profiles for mouse and human erythroid cells refer to RPKM values provided in the GSE53983 submission [34]. ChIPseq signal plots were produced using the Gviz package in R and represent the normalized read density profiles produced by MACS.

## Results

### Binding of GATA1 and PU.1 to the mouse RPS19 proximal promoter region in vitro

**Fig 1:**
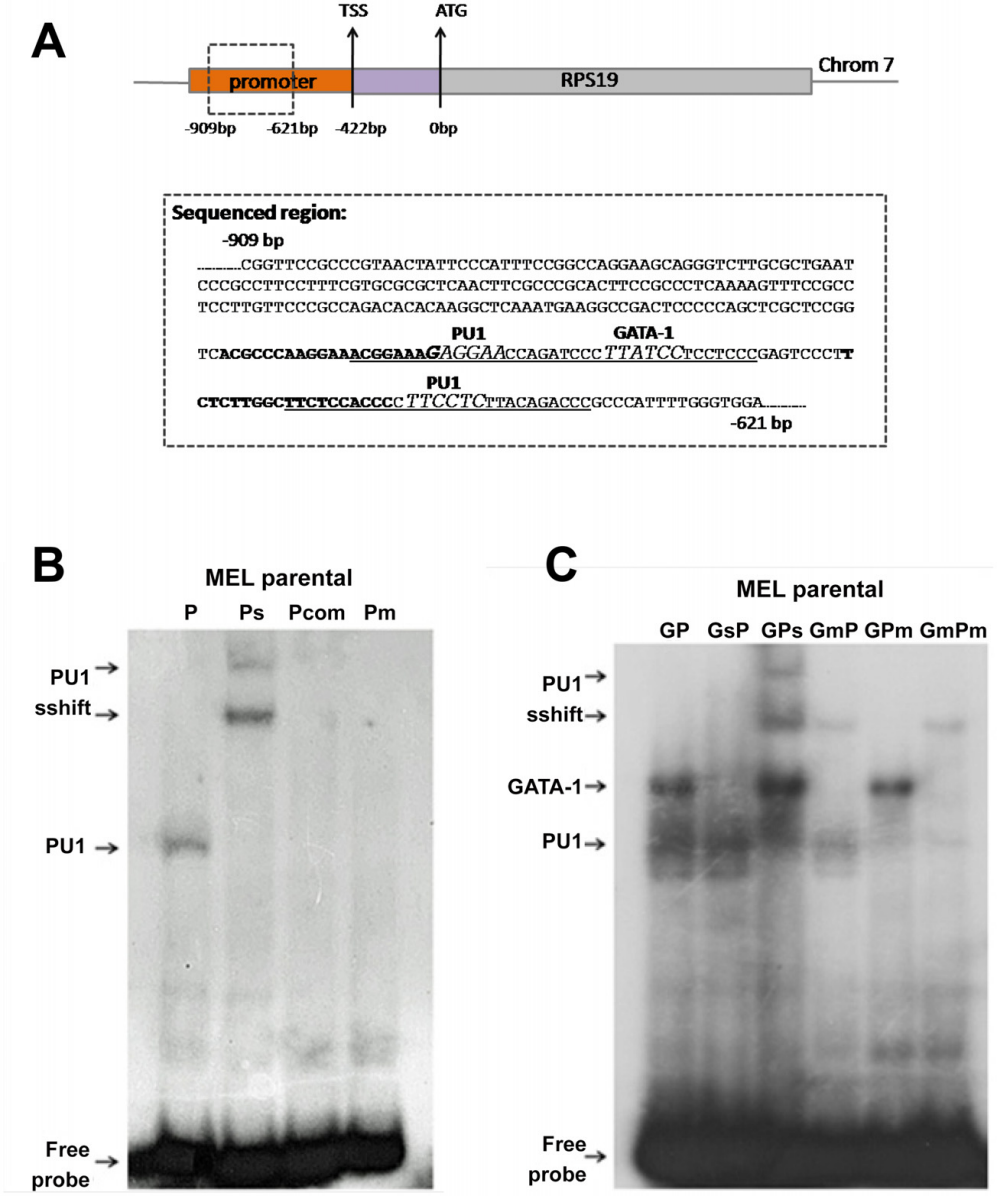
EMSA of GATA1 and PU.1 binding to the proximal promoter region of the mouse RPS19 gene. (A) Schematic representation of the mouse RPS19 gene. The translation initiation codon (ATG) was used to designate the transcription start site (TSS) and the transcription factor (TF) binding site positions. The dashed box upstream of the TSS indicates part of the sequenced RPS19 promoter region that is presented in greater detail below. The sequences for the GP (containing both the GATA1 and PU.1 sites) and P (including only the PU.1 site) EMSA probes are underlined, with the GATA1 and PU.1 consensus binding motifs indicated by italics. RPS19 promoter ChIP primers are indicated in bold. (B) EMSA showing PU.1 binding to the RPS19 promoter region. P: EMSA probe spanning the PU.1 predicted binding motif at position -653 of the RPS19 proximal promoter region. Ps: anti-PU.1 supershifted reaction; Pcom: addition of cold competitor probe; Pm: probe with PU.1 binding site mutated. (C) EMSA showing GATA1 and PU.1 binding to the RPS19 promoter region as two distinct protein complexes. GP: probe spanning the predicted PU.1 and GATA1 binding motifs at position -709bp of the RPS19 proximal promoter region. GsP: anti-GATA supershifted reaction; GPs: anti-PU.1 supershifted reaction; GmP: probe with GATA1 binding site mutated; GPm: probe with PU.1 binding site mutated; GmPm: probe with both GATA1 and PU.1 binding sites mutated.

We initially investigated the mouse RPS19 proximal promoter region experimentally verified from MEL genomic DNA for the presence of consensus binding motifs using standard TF binding prediction tools. This region has been previously investigated by Da Costa and colleagues and shown to be highly conserved between human and mouse [35]. We identified potential PU.1 binding sites at positions -653bp and -709bp upstream of the RPS19 gene translation initiation codon and a potential GATA1 binding site located at position -694bp in close proximity to the PU.1 binding site at -709bp (Fig 1A).

We next tested whether GATA1 and PU.1 could bind to the RPS19 proximal promoter region by EMSA using MEL nuclear extracts. MEL cells are spleen virus-transformed hematopoietic cells blocked at the proerythroblast stage of erythroid differentiation which, upon exposure to inducers such as HMBA, undergo the erythroid differentiation program (Panels A-C in S1 Fig)[36, 37]. MEL cells express both GATA1 and PU.1 in the proliferating non-induced stage; however PU.1 expression is rapidly down-regulated upon MEL cell differentiation (Panel D in S1 Fig)[38]. Therefore, MEL cells represent a suitable model to delineate the relative involvement of GATA1 and PU.1 on the potential regulation of RP genes.

Based on the predicted PU.1 and GATA1 binding motifs in the RPS19 promoter region described above, two different EMSA probes (P and GP) were employed to test TF binding *in vitro*. Binding of PU.1 at position -653bp in probe P was indeed confirmed as the protein complex detected by EMSA (Fig 1B, lane P) was supershifted by an anti-PU.1 antibody (Fig 1B, lane Ps), or abolished by use of a mutated probe (Fig 1B, lane Pm), or by an excess of cold PU.1 competitor (Fig 1B, lane Pcom). Consistent with the rapid PU.1 down-regulation upon MEL cell differentiation (panel E in S1 Fig), PU.1 binding to the RPS19 probe is hardly detectable as early as 6 hours into the induction of MEL erythroid maturation (data not shown).

Probe GP spans the PU.1 and GATA1 sites at positions -709bp and -694bp of the RPS19 gene, respectively. Incubation with MEL nuclear extracts revealed two discrete TF complexes (Fig 1C). Inclusion of an anti-GATA1 (lane GsP) or of an anti-PU.1 (lane GPs) antibody resulted in a diminished GATA1-DNA complex, as presumably the addition of the GATA1 antibody prevented this complex from entering the gel, or a supershifted PU.1 complex, respectively (Fig 1C). Interestingly, the GATA1-specific complex does not appear to diminish when using the anti-PU.1 antibody and *vice versa*, suggesting independent binding of the two TFs to the probe. Furthermore, when using probes mutated for the binding of either factor (Fig 1C, lanes GmP and GPm), loss of binding of the corresponding TF is not accompanied by a concomitant increase in binding of the antagonistic TF. These data confirm that the two MEL nuclear protein complexes binding to the RPS19 proximal promoter motifs contain GATA1 and PU.1, as predicted by our analysis above. PU.1 binding appears to rapidly decline with MEL differentiation, whereas GATA1 binding remains clearly detectable throughout this period (S1E Fig).

### In vivo RPS19 promoter occupancy by PU.1 and GATA1 during MEL cell terminal differentiation

We next assessed GATA1 and PU.1 binding to the RPS19 proximal promoter region *in vivo*, by interrogating a series of ChIPseq data from several models of erythropoiesis [31, 39–41]. GATA1 occupancy of the RPS19 promoter was detected in non-differentiated and DMSO-differentiated MEL cells and in Ter119- mouse fetal liver-derived proerythroblasts, but not in mature Ter119+ fetal liver erythroid cells (panel A in S2 Fig). PU.1 occupancy was also evident in non-induced MEL cells and in mouse embryonic stem cell-derived erythroid progenitors (mES-EPs; panel B in S2 Fig). It should be noted that PU.1 protein levels are very low in mouse fetal liver proerythroblasts, becoming undetectable in mature erythroid cells [42].

**Fig 2:**
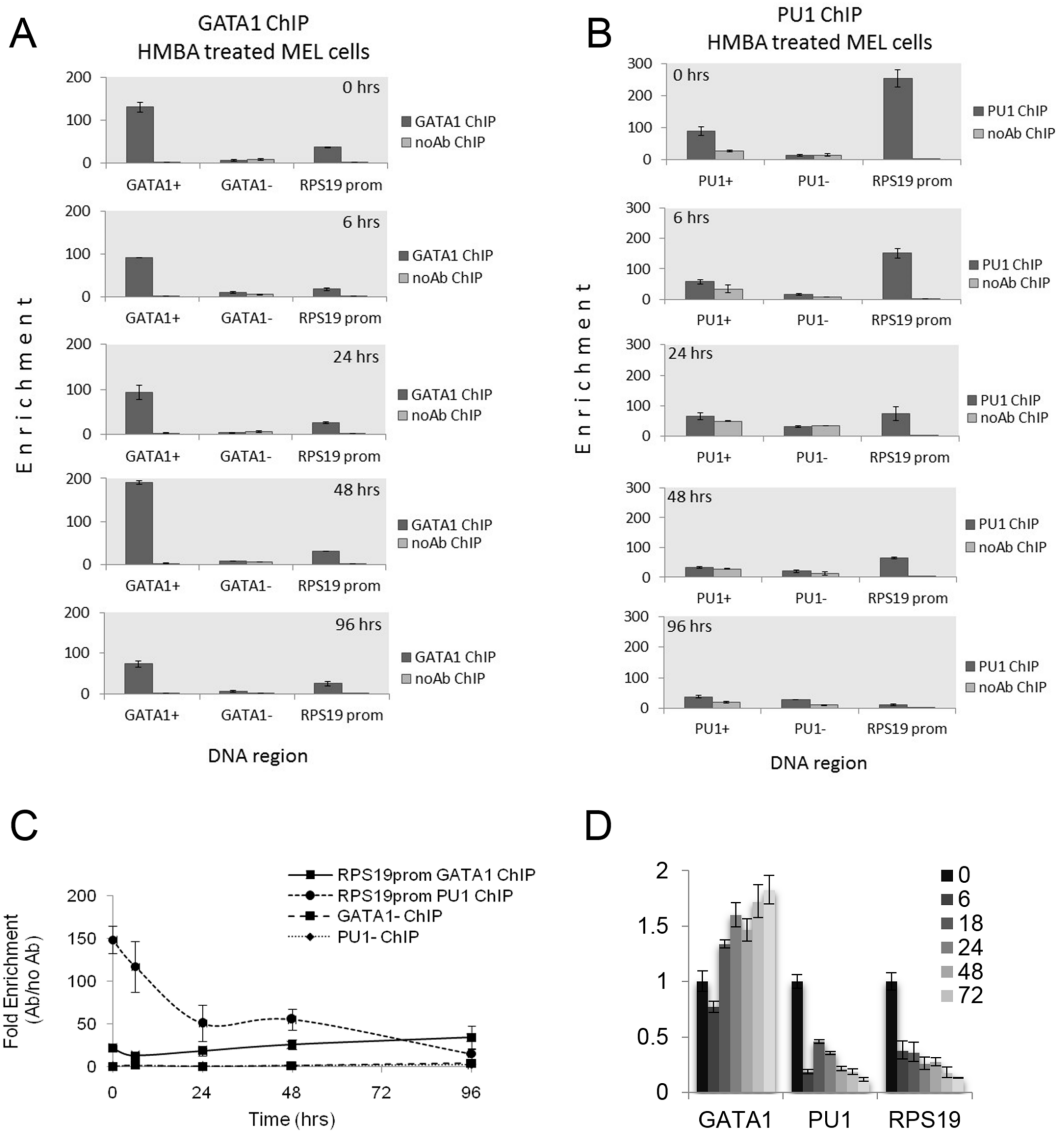
ChIP assays of GATA1 and PU.1 binding to the RPS19 proximal promoter region upon MEL cell differentiation. (A) GATA1 ChIP and (B) PU.1 ChIP of the RPS19 promoter (RPS19 prom) in MEL cells induced to terminally differentiate by treatment with 5mM HMBA. Controls include HS1 in the mouse GATA1 gene locus (GATA1+) and a negative control DNA region (GATA1-) that does not bind GATA1 based on MEL ChIPseq data. PU1+ and PU1- controls include a positive control region (PU.1+) corresponding to the Upstream Regulatory Element (URE) of the PU.1 gene locus and a negative control DNA region (PU.1-) which does not bind PU.1 on the basis of MEL ChIPseq data. No antibody ChIP controls are also shown. (C) Time course of fold-enrichment for GATA1 and PU.1 occupancies of the RPS19 promoter with MEL cell differentiation. Enrichment values for the negative control DNA regions for GATA1 (GATA1- ChIP) and PU.1 (PU1- ChIP) binding are also shown.

The *in vivo* binding of GATA1 and PU.1 to the RPS19 proximal promoter region in erythroid cells at stages where both TFs are expressed, was experimentally validated by ChIP in a time course of HMBA-induced MEL cell differentiation. Enrichment for GATA1 binding was evident in proliferating (0 hours) and in differentiating (6–96 hours) MEL cells (Fig 2A and 2C). GATA1 binding to the RPS19 promoter in MEL cells is not as high as that for the positive control (HS1 of the GATA1 gene locus), it is however well above that obtained for a non-GATA1 binding region of the RPS19 gene locus or the no antibody control and it appears to remain relatively constant during HMBA induced MEL cell differentiation. PU.1 binding was enriched in proliferating MEL cells (0 hours), rapidly declining to near background levels by 96 hours of induction, consistent with the rapid decline in PU.1 protein levels (Fig 2B–2D). At the mRNA level, RPS19 expression declines rapidly by 6 hours of induction, followed by a more gradual decline in later time points (panel D in S1 Fig). These data confirm GATA1 and PU.1 binding to the RPS19 proximal promoter region *in vivo*, with sustained GATA1 binding as PU.1 binding diminishes during MEL differentiation.

### In vivo binding of GATA1 and PU.1 to RP genes implicated in DBA and 5q^-^ syndrome in MEL cells

**Fig 3:**
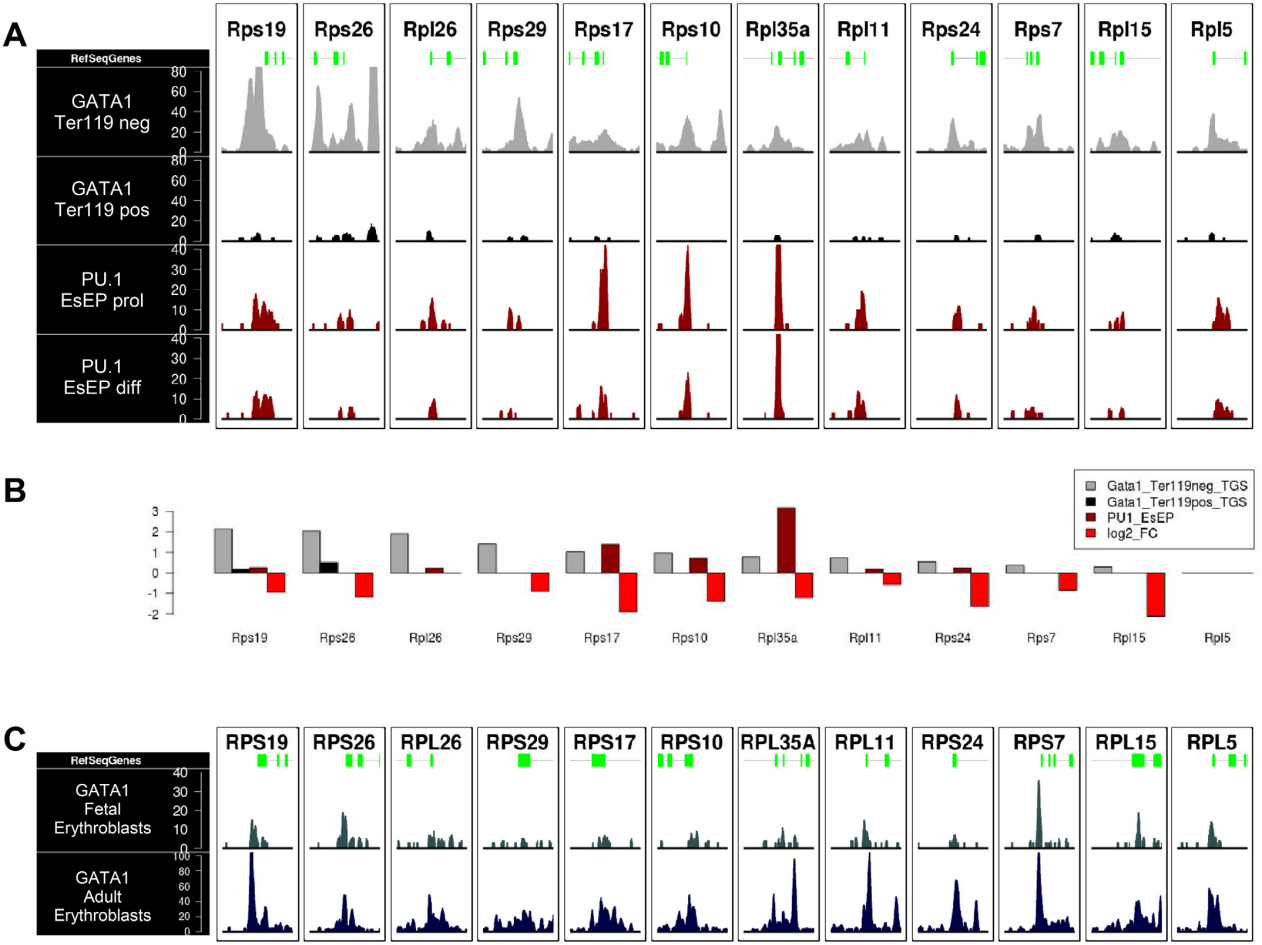
ChIPseq data from mouse and human erythroid models showing GATA1 and PU.1 occupancies of DBA associated RP genes. (A) GATA1 and PU.1 read density profiles in primary mouse fetal liver derived proerythroblasts (negative for Ter119 staining) and mature erythroid cells (positive for Ter119 staining) and in mouse embryonic stem cell-derived proliferating erythroid progenitors (mEs-EPs prol) or mature erythroid cells (mES-EPs diff). Proximal promoter regions of DBA associated ribosomal genes are plotted (±1.5kb around TSS). (B) Quantification of GATA1 and PU.1 occupancies and association with gene expression profiles of DBA associated RPs in Ter119- to Ter119+ maturing mouse fetal liver erythroid cells (normalized number of reads ±10kb around TSS). (C) GATA1 read density profiles of fetal and adult derived primary human erythroid cells of proximal promoter regions of DBA associated RP genes (±1.5kb around TSS).

Our observations on GATA1 and PU.1 binding to the murine RPS19 gene promoter are significant in light of the RPS19 gene being mutated in ~25% of DBA cases. We thus assessed *in silico* whether this was also the case for other mouse homologues of RP genes associated with DBA in man (including RPS14 involved in the 5q^-^ syndrome), by inspecting GATA1 and PU.1 genome occupancy profiles in mouse fetal liver [31] and mEs-EPs [39], respectively (Fig 3A). We found a moderate to high (e.g. RPS19, RPS26 genes) level of GATA1 ChIPseq signal proximally to the TSS regions in the proerythroblast stage (Fig 3A, Ter119- cells), which is lost with terminal differentiation (Fig 3A, Ter119+ cells). Decline of GATA1 binding from these genes during erythroid differentiation correlates with a decrease in their expression levels (Fig 3B, but see also below). PU.1 binding profiles in mEs-EPs generally showed low occupancies except for a strong association with the RPS10, RPS17 and RPL35a genes (Fig 3A).

Due to their clinical relevance, we also surveyed GATA1 occupancies of DBA-associated RP genes in human fetal and adult-derived erythroid cells[43] (Fig 3C). Overall, GATA1 binding profiles in human cells show an extended association with DBA-associated RP gene promoters, particularly in adult erythroid cells compared to the fetal stage. Interestingly, of all DBA-associated RP genes, GATA1 occupancy was the highest in the RPS19 gene in both human and mouse cells. Taken together, these data demonstrate a concordance in GATA1 occupancies of DBA-associated RP genes between mouse and human erythroid cells.

**Fig 4:**
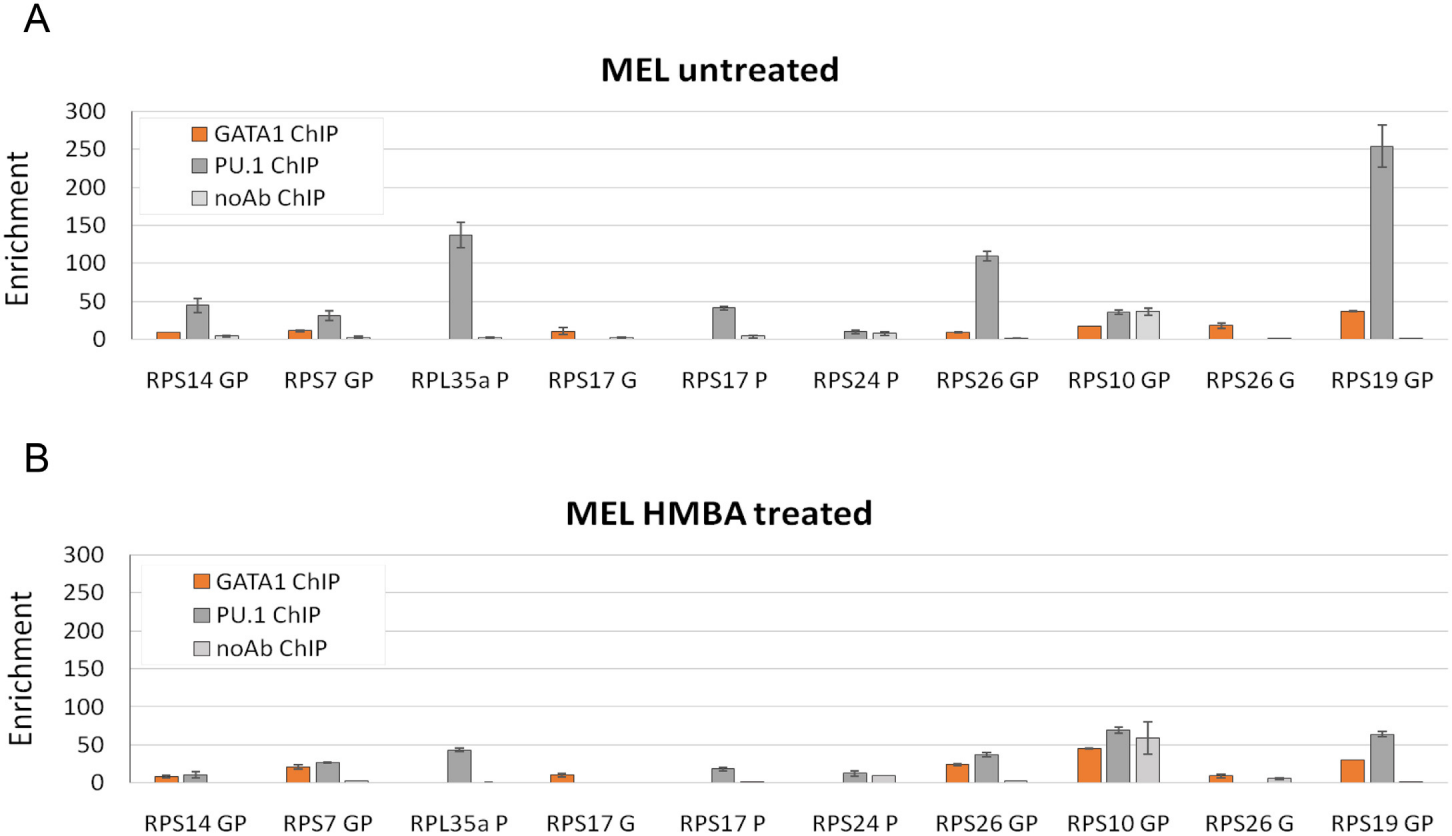
GATA1 and PU.1 ChIP of selected mouse homologues of DBA-associated RP genes in MEL cells. ChIP assays in proliferating (A) and HMBA-differentiated (B) MEL cells. G: ChIP primers for assessing GATA1 binding; P: ChIP primers for assessing PU.1 binding; GP: ChIP primers for assessing GATA1 and PU.1 binding. ChIP primers were designed on the basis of ChIPseq data and their sequences are given in S2 Table.

We next verified by ChIP in MEL cells the GATA1 and PU.1 binding profiles obtained by ChIPseq for selected RP genes associated with DBA and 5q^-^ syndrome (RPS14, RPS7, RPS10, RPS26, RPS17, RPS24 and RPL35a) (Fig 4). The assays agreed with the ChIPseq data in the majority of cases, except for the RPS24 and the RPS10 genes, the latter presenting with high ChIP background levels (Fig 4A and 4B). Interestingly, the RPL35a gene is clearly verified as a PU.1 target in both proliferating and differentiating MEL cells (Fig 4A and 4B), also consistent with the ChIPseq data (Fig 3A). RPS14 and RPS26 are also bound in their promoters by PU.1 and GATA1. Also, GATA1 occupancy was verified in an intronic region of the RPS17 gene locus, whereas PU.1 occupancy was verified in the RPS17 proximal promoter region (Fig 4). Overall, as also seen for RPS19, PU.1 binding to RP target genes is reduced with MEL cell differentiation, whereas GATA1 binding persists in HMBA-differentiated MEL cells (Fig 4B). Expression levels for all RP genes investigated here decline during MEL differentiation (S3 Fig). In conclusion, RP genes associated with DBA and 5q^-^ syndrome were identified as GATA1 and PU.1 targets in human and mouse erythroid cells, suggesting a role for these TFs in RP gene regulation in erythropoiesis.

### GATA1 and PU.1 binding to RP genes during erythropoiesis

**Fig 5:**
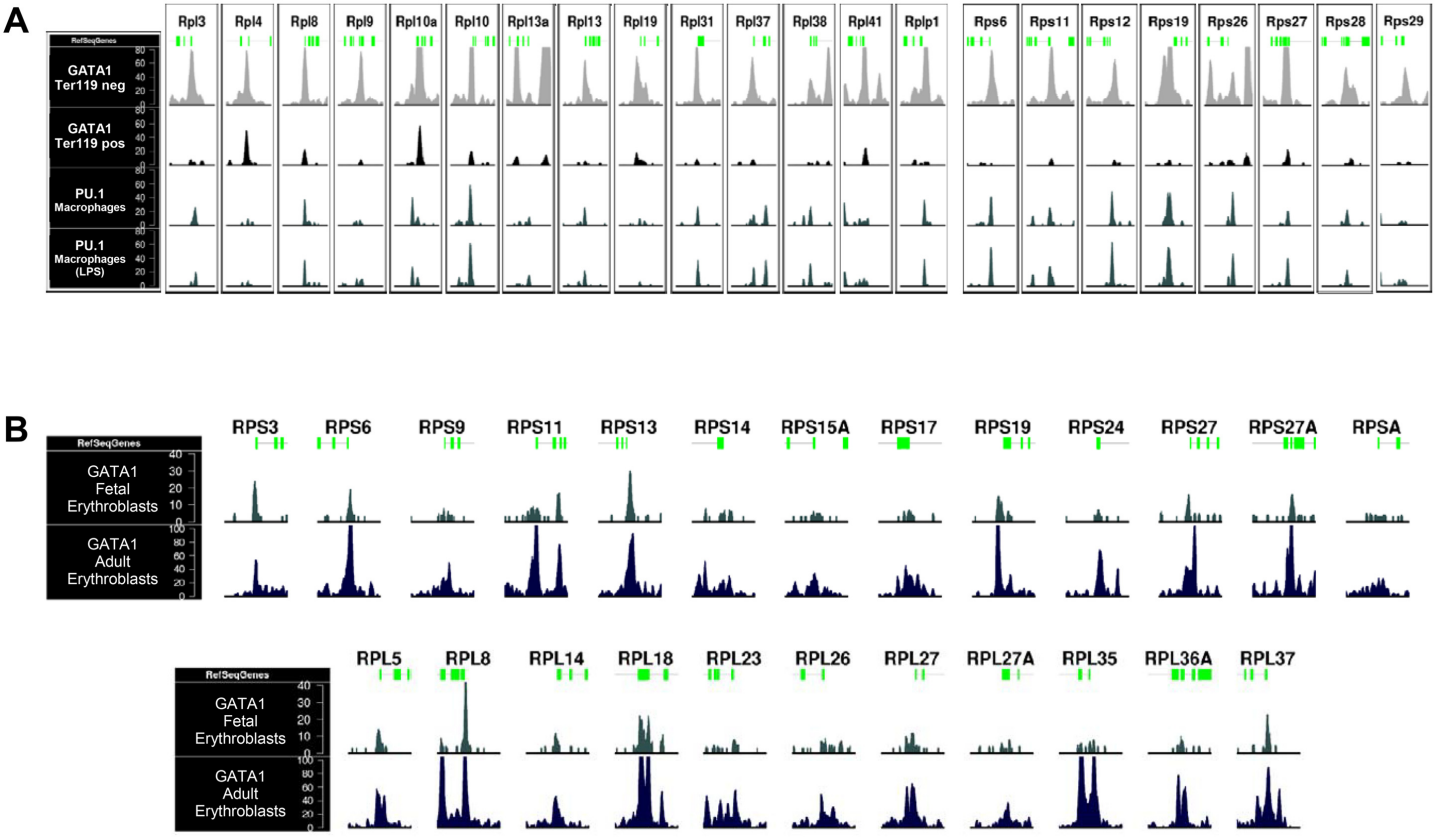
GATA1 and PU.1 ChIPseq occupancies or RP genes in erythroid cells and macrophages. GATA1 occupancies of large and small subunit RP genes (ChIPseq read density profiles, ±1.5kb around TSS is plotted) in (A) primary mouse fetal liver derived proerythroblasts (negative for Ter119 staining) and mature erythroid cells (positive for Ter119 staining)[31] and (B) in human fetal and adult erythroblasts [43]. Note that for comparison, PU.1 occupancies of RP genes in macrophages with or without LPS stimulation are also shown in (A).

We next expanded our *in silico* analysis for GATA1 occupancies to include all RP genes in mouse fetal liver-derived [31] and human primary [43] erythroid cells. From this, it is clear that GATA1 binds to several RP genes in mouse fetal liver erythropoiesis (Fig 5A, S5 Table). GATA1 occupancy within these loci is drastically reduced with terminal maturation, as shown by the absence of GATA1 ChIPseq signal in the Ter119+ fraction of the cells (Figs 3A and 5A). Loss of GATA1 binding from RP genes during erythroid differentiation correlates with a decrease in their expression levels (grey box in S4 Fig). This decline in expression was not accompanied by prominent loss of RNApolII occupancy, or H3K27me3 accumulation in the RP gene loci with erythroid differentiation [31], however we did observe relatively lower levels of the promoter-associated H3K4me3 activation mark and of the H3K79me2 elongation mark (S4 Fig). However, the decline in expression as a result of reduced GATA1 binding could be reflected at the transcript elongation level, as GATA1 has been previously associated with enhanced transcriptional elongation in erythroid cells [44].

In human erythroid cells, several RP genes also appear to be GATA1 targets (Fig 5B). Again, GATA1 RP gene occupancies appear significantly higher in adult erythroid cells compared to fetal cells (Fig 5B), suggesting a developmental aspect to GATA1 RP gene binding and regulation in man. This may be related to the fact that the median age of onset for DBA is two months after birth, at a time when transition to definitive erythropoiesis is completed [45].

We also interrogated ChIPseq data for PU.1 binding profiles in hematopoietic lineages where PU.1 is physiologically active, for example, in macrophages and in lymphoid cells [46]. It is evident that GATA1 and PU.1 occupancies coincided in several, but clearly not all, overlapping RP gene promoters in erythroid cells and in macrophages, respectively (Fig 5A). It is of interest that GATA1 binding to RP genes in erythroid cells appears to be more extensive than that of PU.1 in macrophages. Overall, our findings indicate a clear association of lineage restricted-TF binding with subsets of RP gene loci in different hematopoietic lineages.

### GATA1 binding and RP gene expression levels during terminal erythroid differentiation

**Fig 6:**
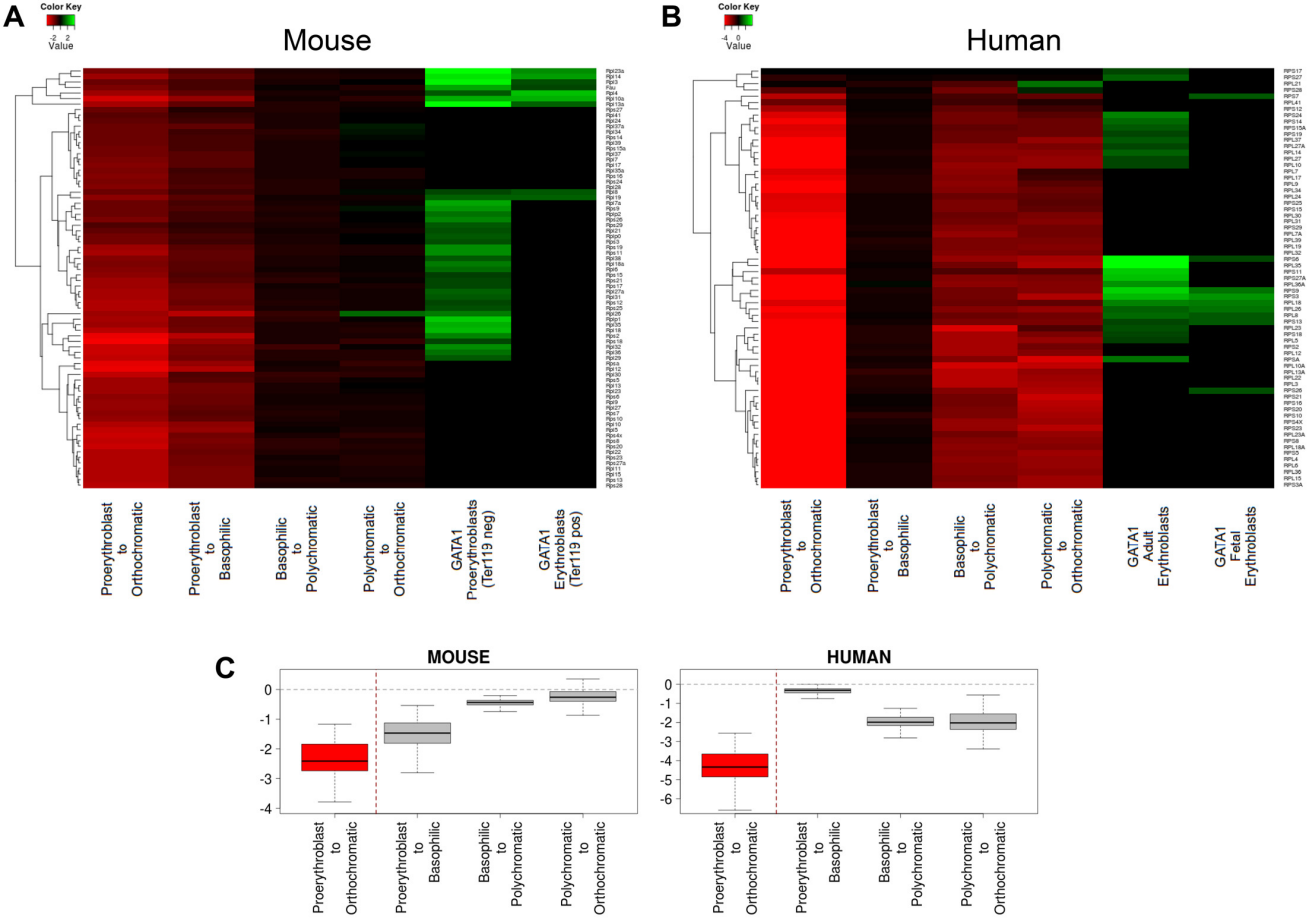
Gene expression fold change profiling of RP genes during human and mouse erythropoiesis in relation to GATA1 occupancy. (A) Gene expression fold change profiling of RP genes during sequential stages of human erythroid terminal differentiation with GATA1 occupancy in fetal and adult derived erythroid cells. (B) Gene expression fold change profiling of RP genes during sequential stages of mouse erythroid terminal differentiation and GATA1 occupancy in early (Ter119-) and late (Ter119+) differentiating mouse fetal liver cells. (C) Box plots of RP gene expression fold change comparing human and mouse differential expression in sequential erythroid differentiation stages, showing an overall steep decline in RP gene expression in early stages of mouse erythroid differentiation compared to human. The different stages of human and mouse erythroid differentiation were FACS purified and subjected to RNAseq as described in An et al.[34].

We next sought to identify the relationship between GATA1 occupancy and RP gene expression profiles during erythroid differentiation by analyzing RNAseq data of sequential erythroid differentiation stages in mouse and human cells [34]. We find that expression of the majority of RP genes is drastically down-regulated with the onset of erythroid differentiation in both human and mouse (Fig 6A and 6B), however a stage-by-stage comparison reveals differences in their timing. Specifically, RP gene expression is highly down-regulated in the early stages (proerythroblast to basophilic) of mouse erythropoiesis, whereas in humans RP gene down-regulation occurs in later, more differentiated stages (basophilic to polychromatic) (Fig 6C). These data further highlight differences between human and murine erythropoiesis, which may also account for the relative inefficiency of mouse models engineered for RP gene mutations to fully recapitulate the DBA phenotype.

**Fig 7:**
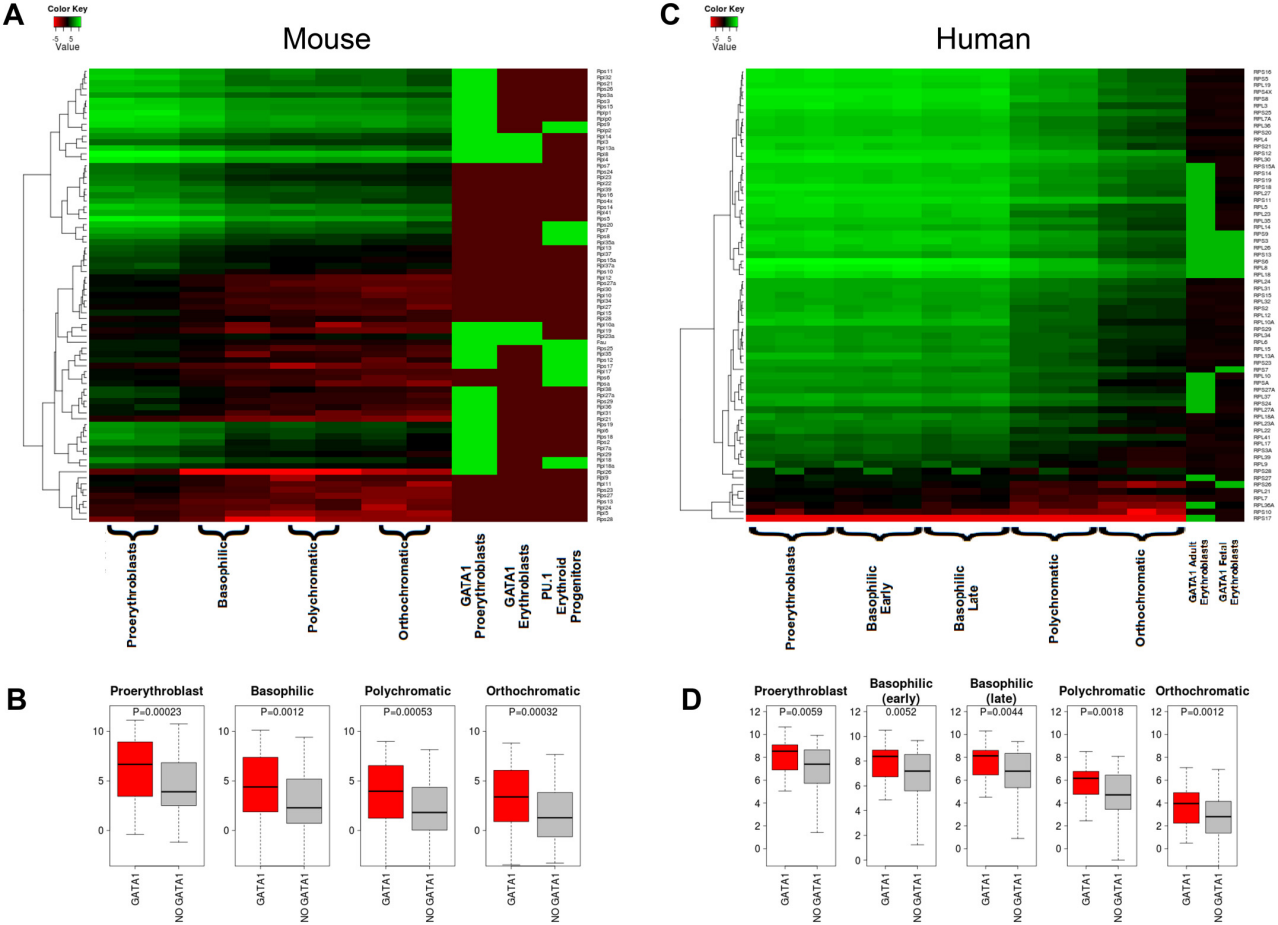
Correlation of GATA1 occupancy with absolute RP expression levels in distinct stages of mouse and human terminal erythropoiesis. (A) The absolute mRNA expression levels of RP genes in morphologically distinct stages of mouse terminal erythroid differentiation are shown. GATA1 occupancy in early (Ter119-) and late (Ter119+) erythroid differentiation stages and PU.1 occupancy in mEsEPs cells is also included. (B) The quantification of absolute mRNA levels between GATA1 occupied (red) and non-GATA1 occupied (grey) RP genes consistently shows a significant association of GATA1 binding with higher expressed RP genes (red), despite the overall decline in RP gene expression with erythroid differentiation (grey). (C) The absolute mRNA expression levels of RPs in morphologically distinct stages of human terminal erythroid differentiation and GATA1 occupancy in fetal and adult derived human erythroid cells is shown. (D) The quantification of absolute mRNA levels between GATA1 occupied (red) and non occupied (grey) RPs again shows a consistently significant association of GATA1 binding with higher expressed RP genes (red), despite the overall decline in RP gene expression with erythroid differentiation (grey).

Lastly, we quantified the absolute expression levels of GATA1-occupied RP genes. This revealed a clear association of GATA1 binding with the higher expressing RP genes. This is maintained throughout erythroid differentiation and is evident in both mouse (Fig 7A and 7B) and human (Fig 7C and 7D). Whereas it is not clear from this analysis if GATA1 is actively involved in the establishment of the differential expression levels of RP genes, we note the cross-species conservation of its association with higher expressing RP genes.

## Discussion

Haploinsufficiency in specific RP genes perturbing balanced ribosome biogenesis, invariably results in specific hematopoietic disorders such as the 5q^-^ syndrome, isolated congenital asplenia and DBA [8, 47]. The latter is characterized by a very specific phenotype of erythroid hypoplasia in the bone marrow, highlighting an exceptional requirement for RP balanced production in terminal erythroid differentiation [7, 48]. Recent data have connected the essential, erythropoietic GATA1 TF to DBA [48, 49]. First, rare mutations in the GATA1 gene resulting in the production of a shorter, N-terminally truncated GATA1 protein (GATA1s) were identified in DBA patients with no detectable RP gene defects [22, 23]. In addition, RP gene haploinsufficiency was recently shown to lead to inefficient translation of the long isoform of GATA1 in proerythroblasts [50], which in turn results in increased apoptosis and impaired erythroid maturation. These findings link RP gene haploinsufficiency to defects in GATA1 functions in regulating erythropoiesis [51, 52]. However, the possibility that GATA1 directly regulates RP gene expression in erythroid cells has not been systematically addressed. In fact, RP gene transcriptional regulation in general remains poorly characterized [5].

Here, we asked whether lineage-specific hematopoietic TFs might be involved in RP gene regulation in erythroid cells. We initially used a computational approach to identify consensus binding motifs for the key hematopoietic TFs GATA1 and PU.1 in a region upstream of the start codon of the murine RPS19, at positions -694bp and -709bp. We verified by EMSA *in vitro* binding of GATA1 and PU.1 to these motifs in nuclear extracts from MEL cells. Furthermore, we confirmed these findings *in vivo* by ChIP assays using chromatin from MEL cells, further supported by ChIPseq data in mouse fetal liver erythropoiesis. We next used publically available ChIPseq data to extend our observations of GATA1 and/or PU.1 erythroid-specific occupancies to many additional RP genes and, moreover, we confirmed by ChIP the GATA1 and/or PU.1 binding in MEL chromatin for a subset of RP genes that are mutated in DBA. Importantly, our observations were also extended to human erythropoiesis where we observe higher GATA1 occupancies of RP genes in the adult compared to the fetal stage of erythropoiesis. We further describe a difference in the timing of RP gene transcriptional decline in mouse versus human erythroid differentiation, in that this appears to take place at a later differentiation stage in human cells. Lastly, we find that despite the overall decline in RP gene expression levels, GATA1 binding is associated with higher expressed RP genes throughout erythroid terminal differentiation stages in human and in mouse.

The GATA1 and PU.1 binding sites upstream of the RPS19 promoter that we describe here coincide with a region of high sequence conservation between the mouse and human Rps19 genes previously described by Da Costa et al.[35]. The clustered PU.1 and GATA1 motifs (Fig 1A) are conserved between mouse and human, whereas the second, more downstream PU.1 site is not (not shown). ChIPseq data show the presence of two GATA1 peaks in the mouse RPS19 upstream promoter region, suggesting that it may bind to both regions (Fig 3A, panel A in S2A Fig). Analysis of GATA1 ChIPseq data in human erythroid cells shows the presence of one strong binding peak upstream of the RPS19 promoter. This peak spans a sequence that is 62% identical between human and mouse and it includes the homologous PU.1 and GATA1 motifs that we identified in the mouse sequence (not shown). In addition, this region falls within a block of high information content (BHIC) in a multi-species alignment of the RPS19 extended genomic locus [53], further supporting a regulatory role for these sequences in RPS19 expression.

By expanding our studies to include all RP genes using publicly available ChIPseq data in erythroid cells (S4 Table), we found that GATA1 and, to a lesser extent, PU.1 bind to the majority of RP genes in erythroid cells (S5 Table). Interestingly, PU.1 occupancy of RP genes is also clearly observed in macrophages (Fig 5A), with an almost complete overlap with PU.1 RP gene occupancies in erythroid cells. These data clearly establish RP genes as PU.1 targets in hematopoiesis extending beyond the erythroid lineage. Importantly, ribosome biosynthesis genes have been reported previously as being GATA1 and PU.1 targets in erythroid cells [31, 54], further supporting the involvement of these TFs in controlling ribosome biogenesis.

How might PU.1 and GATA1 binding serve to regulate RP gene expression in erythroid cells? Recent observations suggest that PU.1 acts primarily as an activator of non-erythroid genes in proliferating erythroid progenitors [39, 40]. Thus, we propose that PU.1 maintains RP gene expression in immature erythroid cells. As PU.1 has been reported to bind to common subsets of genes in different hematopoietic lineages [39], it is likely that PU.1 functions in RP gene activation in immature erythroid cells are conserved in other hematopoietic lineages, such as macrophages. As cells commit to terminal erythroid differentiation, GATA1 occupies target RP genes which are not completely overlapping with those occupied by PU.1 in erythroid progenitors. Interestingly, GATA1 occupancy of RP genes in immature erythroid cells is reduced with terminal erythroid differentiation (Fig 5A). This could be due to the global condensation of chromatin that takes place with erythroid maturation, which could result in reduced access of the GATA1 antibody to its epitope in crosslinked chromatin in maturing erythroid cells, and/or in disengagement of GATA1 protein from chromatin as this undergoes condensation. Regardless, we do find a clear correlation of GATA1 occupancy with relatively higher absolute expression of target RP genes compared to non-GATA1 target RP genes throughout terminal erythroid differentiation in mouse and human (Figs 6 and 7), suggesting an activating role for GATA1 in this context. It may be possible that GATA1 binding in immature erythroid cells serves to fine tune expression of a subset of RP genes in preparation for the high demands of hemoglobin synthesis. This scenario may be in line with the concept of “specialized ribosomes” having a unique composition or activity in different tissues [55, 56]. Taken together, we propose a model whereby PU.1 serves as an activator of a subset of RP genes in early erythroid progenitors (and potentially in earlier stages of hematopoiesis and in other lineages). Upon commitment to terminal erythroid differentiation, as PU.1 expression is repressed by GATA1, the latter takes over the regulation of a non-overlapping subset of RP genes long enough to sustain massive hemoglobin synthesis against a backdrop of global RP gene down-regulation.

Our data on the dynamic GATA1 occupancies of RP genes in erythroid cells raise the prospect of GATA1 directly regulating RP gene expression in the erythroid lineage, thus providing an additional mechanism for GATA1’s implication in DBA. It also raises the prospect that mutations in TF binding motifs in RP gene regulatory regions, which are not routinely screened in diagnosis, may be an alternative underlying cause of DBA. According to our model, mutations in GATA1 (or PU.1) binding motifs in RP gene regulatory elements resulting in reduced RP gene expression and/or mutations resulting in the production of GATA1s which may be deficient in properly regulating RP gene expression in erythroid differentiation, would lead to RP imbalance and disease [52]. This model also raises the intriguing possibility that full length GATA1 can promote its self-translation during terminal erythroid differentiation by directly regulating the expression of components of the translational machinery.

## Supporting Information

S1 Fig Analysis of HMBA induced MEL cell differentiation. MEL cells grown in culture for 96 hr in the presence of HMBA (5mM) were assessed for their proliferation potential (A), cell viability (B), and differentiation capacity (accumulation of benzidine-positive stained hemoglobin-containing cells) (C) as described in Materials and Methods. (D) The expression profiles of β^major^ globin, GATA1, PU.1, and RPS19 genes in HMBA-induced MEL cell differentiation were analyzed by qRT-PCR analysis. (E) Western blot analysis for GATA1 and PU.1 protein levels in nuclear extracts isolated from either untreated or HMBA-treated MEL cells. Nucleophosmin was used as protein loading control.
(PPT)

S2 Fig ChIPseq peaks of GATA1 and PU.1 binding to RPS19. (A) GATA1 binding profiles in the RPS19 gene locus in proliferating and DMSO-induced MEL cells as well as in fetal liver derived Ter119- and Ter119+ immature and mature erythroid cells, respectively. (B) PU.1 binding profiles by ChIPseq to the RPS19 gene locus in two independent experiments (A or B) using differentiated MEL cells or mES-EPs [39].
(PPT)

S3 Fig Steady-state mRNA levels of various mouse homologues of DBA-related RP genes during the course of MEL cell differentiation. (PPT)

S4 Fig Variation in gene expression levels, GATA1 occupancy, H3K4me3 activation mark and H3K79me2 elongating mark. (A) mouse homologues of DBA associated or (B) all RP genes in mouse fetal liver derived Ter119- erythroblasts and Ter119+ mature erythroid cells. P value was calculated using the one sided Wilcoxon rank sum test.
(PPT)

S1 Table EMSA probes used in the in vitro binding assays. Only the forward single stranded oligonucleotide is depicted. TF binding sites appear underlined. Mutations introduced to abolish TF binding appear as bold letters.
(DOC)

S2 Table List of ChIP primers used in this study. (DOC)

S3 Table List of qRT-PCR primers used in this study. (DOC)

S4 Table List of NGS datasets used in this study. (DOC)

S5 Table Total gene scores (TGS) of GATA1 or PU.1 binding to all RP genes and selected GATA1 target genes in mouse fetal liver erythroid cells and in mES-EPs. Indicated by asterisk are the RP genes that have been found mutated in DBA.
(DOC)
