# Supplementary figures and images for: GATA1 and PU.1 Bind to Ribosomal Protein Genes in Erythroid Cells: Implications for Ribosomopathies
Source: PLoS One. 2015 Oct 8;10(10):e0140077. doi: 10.1371/journal.pone.0140077 (PMC4598024; doi:10.1371/journal.pone.0140077)

## Slide 1
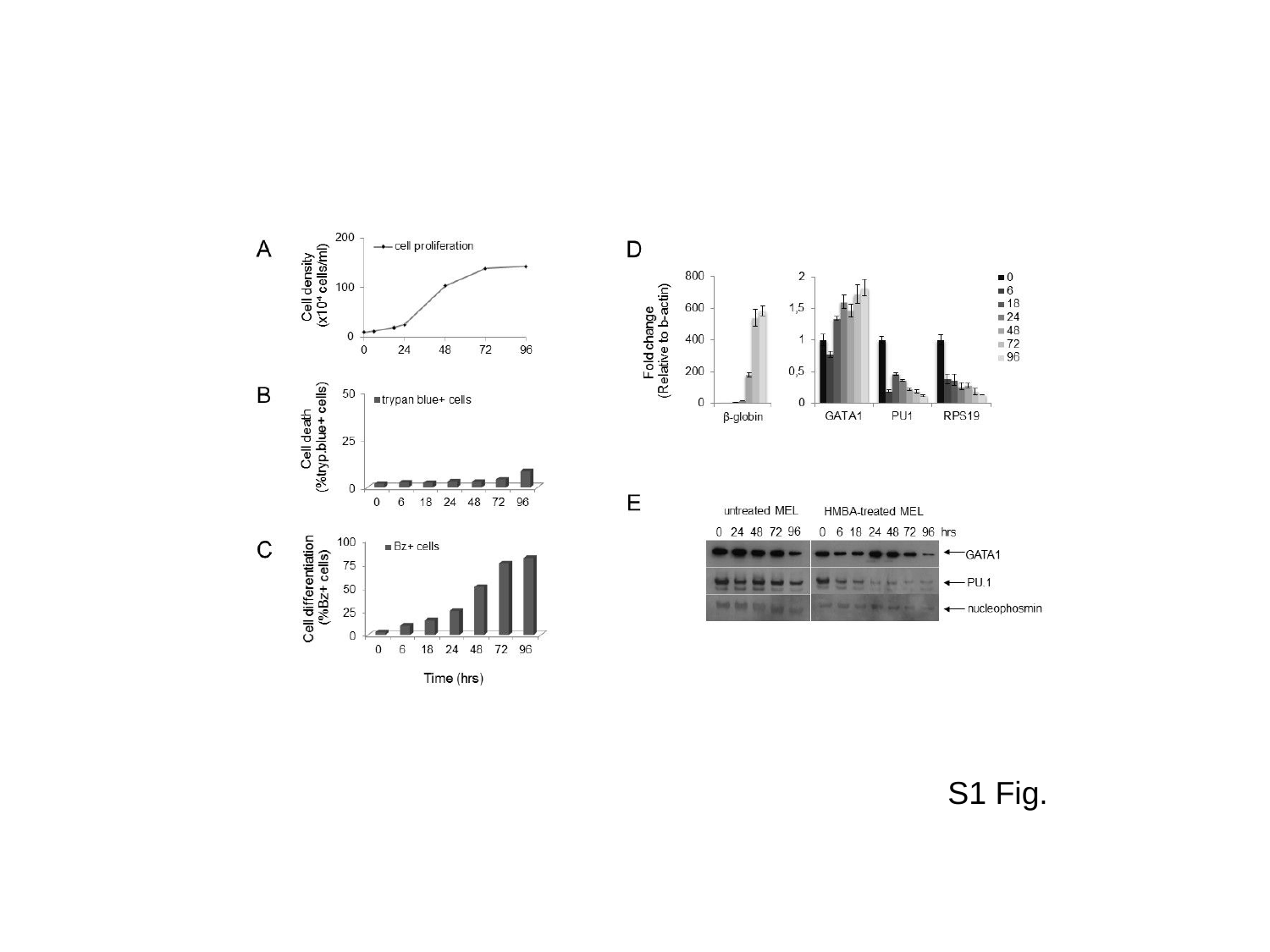

S1 Fig.

Supplement: S1 Fig — MEL cells grown in culture for 96 hr in the presence of HMBA (5mM) were assessed for their proliferation potential (A), cell viability (B), and differentiation capacity (accumulation of benzidine-positive stained hemoglobin-containing cells) (C) as described in Materials and Methods. (D) The expression profiles of βmajor globin, GATA1, PU.1, and RPS19 genes in HMBA-induced MEL cell differentiation were analyzed by qRT-PCR analysis. (E) Western blot analysis for GATA1 and PU.1 protein levels in nuclear extracts isolated from either untreated or HMBA-treated MEL cells. Nucleophosmin was used as protein loading control. (PPT) [file pone.0140077.s001.ppt]

## Slide 1
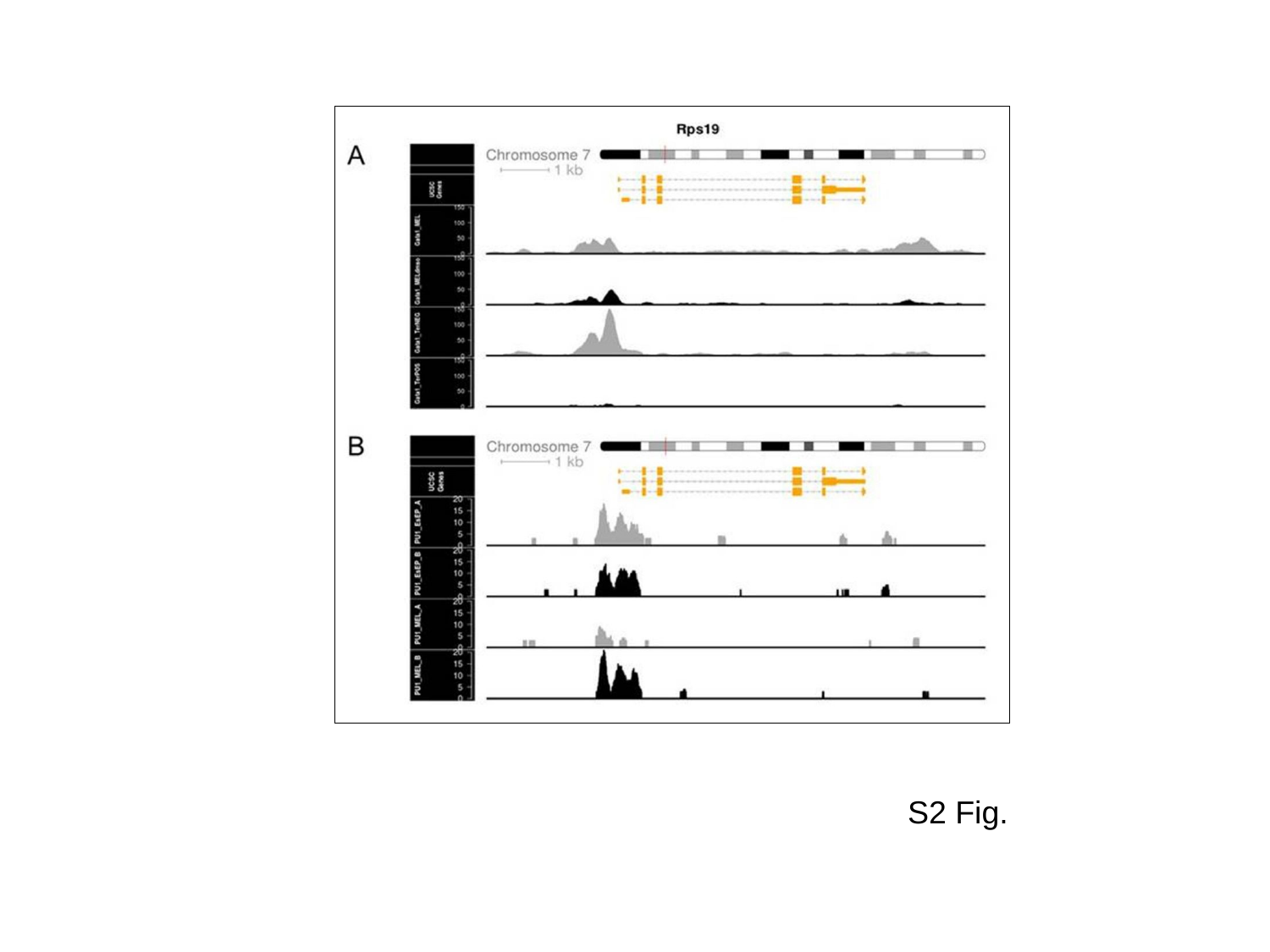

S2 Fig.

Supplement: S2 Fig — (A) GATA1 binding profiles in the RPS19 gene locus in proliferating and DMSO-induced MEL cells as well as in fetal liver derived Ter119- and Ter119+ immature and mature erythroid cells, respectively. (B) PU.1 binding profiles by ChIPseq to the RPS19 gene locus in two independent experiments (A or B) using differentiated MEL cells or mES-EPs [39]. (PPT) [file pone.0140077.s002.ppt]

## Slide 1
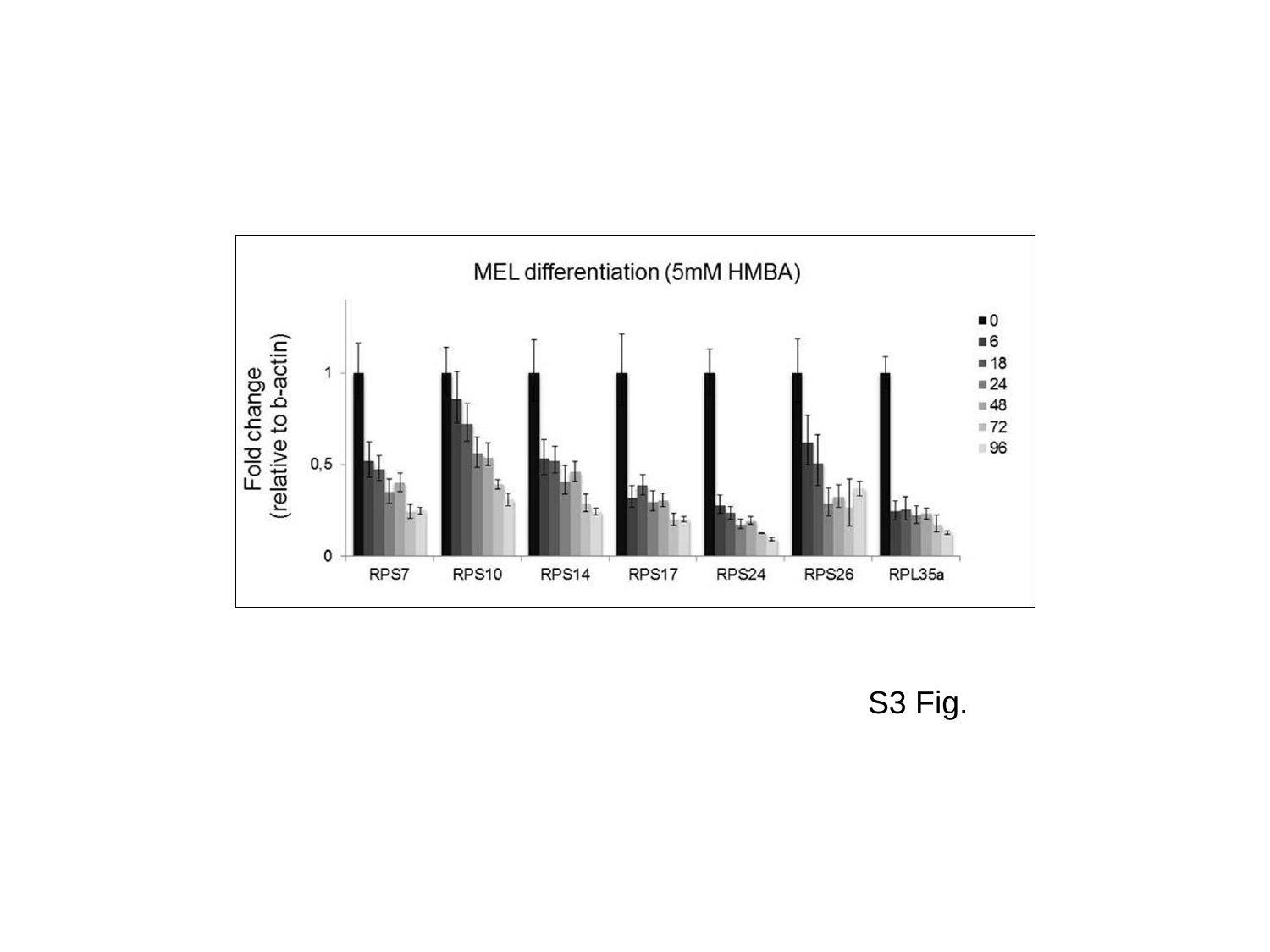

S3 Fig.

Supplement: S3 Fig — (PPT) [file pone.0140077.s003.ppt]

## Slide 1
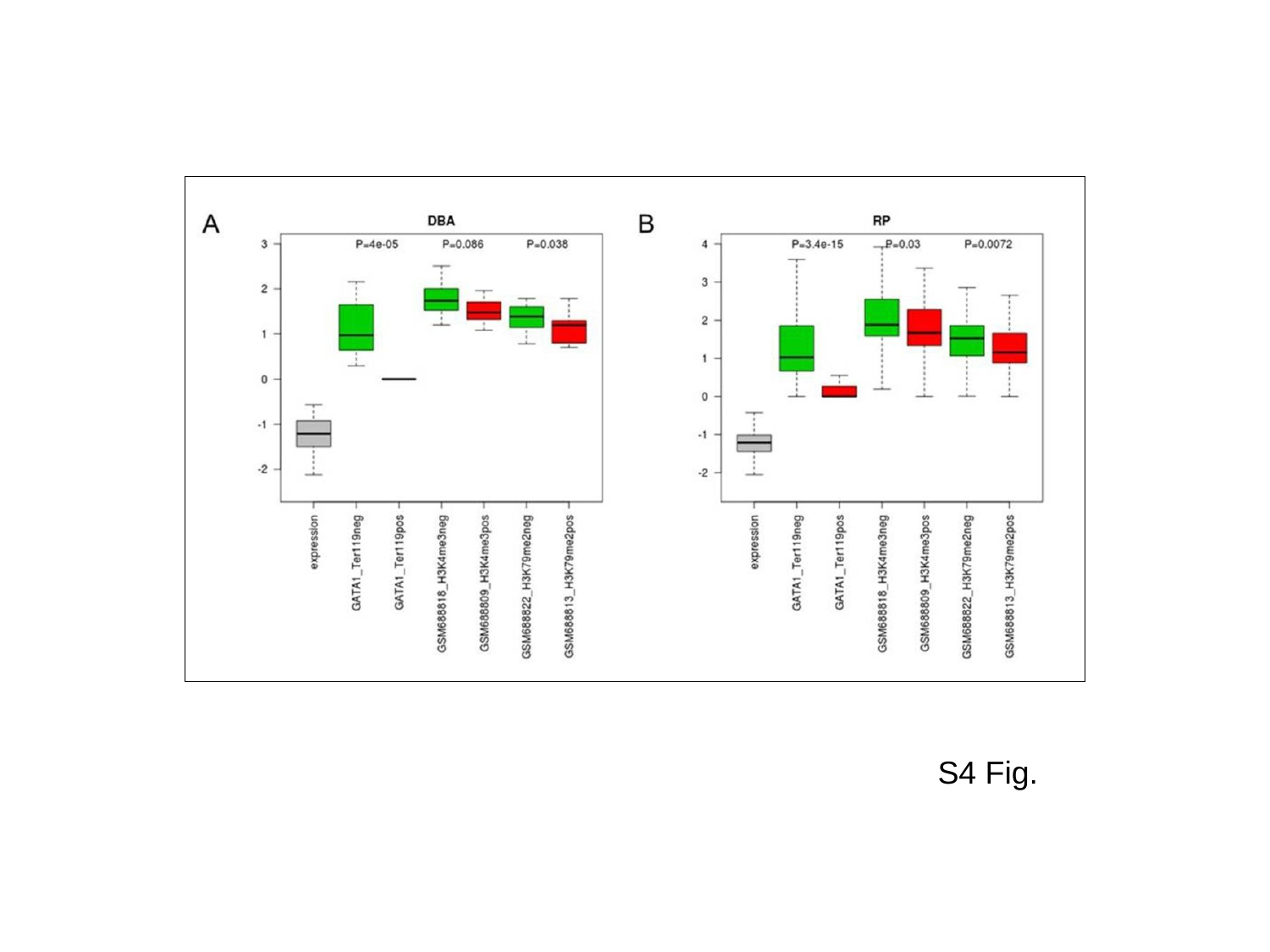

S4 Fig.

Supplement: S4 Fig — (A) mouse homologues of DBA associated or (B) all RP genes in mouse fetal liver derived Ter119- erythroblasts and Ter119+ mature erythroid cells. P value was calculated using the one sided Wilcoxon rank sum test. (PPT) [file pone.0140077.s004.ppt]
