# Supplementary material for: GATA1 and PU.1 Bind to Ribosomal Protein Genes in Erythroid Cells: Implications for Ribosomopathies
Source: PLoS One. 2015 Oct 8;10(10):e0140077. doi: 10.1371/journal.pone.0140077 (PMC4598024; doi:10.1371/journal.pone.0140077)
Supplement: S1 Table — Only the forward single stranded oligonucleotide is depicted. TF binding sites appear underlined. Mutations introduced to abolish TF binding appear as bold letters. (DOC) [file pone.0140077.s005.doc]

**S1 Table**

| **EMSA probe** | **Sequence** |
| --- | --- |
| P | 5’-CTTCTCCACCCCTTCCTCTTACAGACCCGCCC-3’ |
| Pm | 5’-GCTTCTCCACCCCT**CT**CTCTTACAGACCCGCCC-3’ |
| Pcom | 5’-TAACCTCTGAAAGAGGAACTTGGT-3’ |
| GP | 5’-ACGGAAAGAGGAACCAGATCCCTTATCCTCCTCCC- 3’ |
| GmP | 5’-ACGGAAAGAGGAACCAGATCCCTT**CG**CCTCCTCCC- 3’ |
| GPm | 5’-ACGGAAAGAG**AG**ACCAGATCCCTTATCCTCCTCCC-3’ |
| GmPm | 5’-ACGGAAAGAG**AG**ACCAGATCCCTT**CG**CCTCCTCCC-3’ |
