# Supplementary material for: GATA1 and PU.1 Bind to Ribosomal Protein Genes in Erythroid Cells: Implications for Ribosomopathies
Source: PLoS One. 2015 Oct 8;10(10):e0140077. doi: 10.1371/journal.pone.0140077 (PMC4598024; doi:10.1371/journal.pone.0140077)
Supplement: S2 Table — (DOC) [file pone.0140077.s006.doc]

**S2** Table

| **ChIP primer** | **Primer Sequence** |
| --- | --- |
| RPS19prom F | 5’- ACGCCCAAGGAAACGGAAAG -3’ |
| RPS19prom R | 5’- GGGTGGAGAAGCCAAGAGAA -3’ |
| GATA+ F | 5’- TCAGGGAAGGATCCAAGGAA -3’ |
| GATA+ R | 5’- CCGGGTGTAAGCGTCTTCT -3’ |
| GATA- F | 5’- CACTAGCAGCTGGGTGGGTTA -3’ |
| GATA- R | 5’- TGCCGCTTGCCTTTGTAAG -3’ |
| PU1+ F | 5’- GGGAGGCAGAGCACACATG -3’ |
| PU1+ R | 5’- GTTTCCACATCGGCAGCAG -3’ |
| PU1- F | 5’- GCATCTGGTGGGTGGACAAG -3’ |
| PU1- R | 5’- GCGCGCCATCTTCTGGTA -3’ |
| GAPDH F | 5’- CCAATGTGTCCCGTCGTGGATCT -3’ |
| GAPDH R | 5’- GTTGAAGTCGCAGGAGACAAC -3’ |
| RPS14GP F | 5’- TAGGCAGATTCGGTCACAGCAA -3’ |
| RPS14GP R | 5’- TCACGGTTTCCAGCGAAGCTAA -3’ |
| RPS10GP F | 5’- CGGATGGCGACAGATGGAGC -3’ |
| RPS10GP R | 5’- TTCCTTTCCAGCCTCCGCCT -3’ |
| RPS7GP F | 5’- ATATCCGGGTGGCGGAAGAAA -3’ |
| RPS7GP R | 5’- TTTCCGGTACACTCTAGGCGGT -3’ |
| RPS26GP F | 5’- TCCAGAGCGGAAGTTATCCA -3’ |
| RPS26GP R | 5’- CAACTCCCACAATGCATCTC -3’ |
| RPS17G F | 5’- ACCCGTAAGATGGCTCAACTGT -3’ |
| RPS17G R | 5’- AGCTCTGGCTGTTACCTGCTTT -3’ |
| RPS26G F | 5’- ACCCAGCCATATCTCTCCTTGG -3’ |
| RPS26G R | 5’- GGAGCAGCAATGAGCGTGTC -3’ |
| RPL35aP F | 5’- TGCTTTCTGGCGCAGTGTGA -3’ |
| RPL35aP R | 5’- ATGGCGGAAAGAGGAAGGCAA -3’ |
| RPS17P F | 5’- ATGTTGGCGGGTCTCGGTAAA -3’ |
| RPS17P R | 5’- GCTTTCTGGCCGCGCTTTAT -3’ |
| RPS24P F | 5’- TCCTCGGGCTCTCAGCACTAT -3’ |
| RPS24P R | 5’- ATGGCTGCAGCTCTAGTGGG -3’ |
