# Supplementary material for: GATA1 and PU.1 Bind to Ribosomal Protein Genes in Erythroid Cells: Implications for Ribosomopathies
Source: PLoS One. 2015 Oct 8;10(10):e0140077. doi: 10.1371/journal.pone.0140077 (PMC4598024; doi:10.1371/journal.pone.0140077)
Supplement: S3 Table — (DOC) [file pone.0140077.s007.doc]

**S3** Table

| **qPCR Primer** | **Primer Sequence** |
| --- | --- |
| b-actin F | 5’- CTAAGGCCAACCGTGAAAAG -3’ |
| b-actin R | 5’- ATCACAATGCCTGTGGTACG -3’ |
| HBB-b1 F | 5’- ATGGCCTGAATCACTTGGAC -3’ |
| HBB-b1 R | 5’- ACGATCATATTGCCCAGGAG -3’ |
| GATA1 F | 5’- TCAAGCTCCATCAGGTGAACC -3’ |
| GATA1 R | 5’- TTTTCCCTTTGCCAGATGCC -3’ |
| PU1 F | 5’- ACAGGCGTGCAAAATGGAAG -3’ |
| PU1 R | 5’- AGTCATGCATTGGACGTTGG -3’ |
| RPS19 F | 5’- GGGCTGAAAATGGTGGAAAAGG -3’ |
| RPS19 R | 5’- ATGCTTCTTGTTGGCAGCTG -3’ |
| RPS14 F | 5’- TCTGCCACATCTTTGCATCC -3’ |
| RPS14 R | 5’- ACATGGCTGCATATGGAGAGG -3’ |
| RPS7 F | 5’- AAGCACGTGGTCTTCATTGC -3’ |
| RPS7 R | 5’- GGGGCGCTTCTGCTTATTTTTC -3’ |
| RPS10 F | 5’- TCGCCAAAAAGGATGTCCAC -3’ |
| RPS10 R | 5’- TCGAGACTTGAGAGACTGCATG -3’ |
| RPS17 F | 5’- ACCAAGGAGATGCTGAAGCTC -3’ |
| RPS17 R | 5’- TTAAACAGCTCCACGTGGTG -3’ |
| RPS24 F | 5’- AAAGACCTCCCGAAAACAGC -3’ |
| RPS24 R | 5’- TCACCGCAGATCTACTCCTTTG -3’ |
| RPS26 F | 5’- ATCTGAAGCAAGCGTCTTCG -3’ |
| RPS26 R | 5’- ACAACCTTGCTATGGATGGC -3’ |
| RPL35a F | 5’- AAACAGCGGTATGGTTCGTG -3’ |
| RPL35a R | 5’- AATCCGGGATGGGTACAGC -3’ |
