# Supplementary material for: GATA1 and PU.1 Bind to Ribosomal Protein Genes in Erythroid Cells: Implications for Ribosomopathies
Source: PLoS One. 2015 Oct 8;10(10):e0140077. doi: 10.1371/journal.pone.0140077 (PMC4598024; doi:10.1371/journal.pone.0140077)
Supplement: S4 Table — (DOC) [file pone.0140077.s008.doc]

**S4 Table**

| **NGS Dataset** | **Cell Type** | **Species** | **Accession** | **Reference (PMID)** |
| --- | --- | --- | --- | --- |
| GATA1 | Ter119neg | Mouse | PRJEB1476 | 23519611 |
| GATA1 | Ter119pos | Mouse | PRJEB1476 | 23519611 |
| PU1 | EsEP | Mouse | GSE21953 | 21695229 |
| PU1 | Macrophages | Mouse | GSE38377 | 23332752 |
| PU1 | Macrophages + LPS | Mouse | GSE38377 | 23332752 |
| H3K4me3 | Ter119neg | Mouse | GSE27893 | 21860024 |
| H3K4me3 | Ter119pos | Mouse | GSE27893 | 21860024 |
| H3K79me2 | Ter119neg | Mouse | GSE27893 | 21860024 |
| H3K79me2 | Ter119pos | Mouse | GSE27893 | 21860024 |
| GATA1 | Fetal Erythroblasts | Human | GSE36994 | 23041383 |
| GATA1 | Adult Erythroblasts | Human | GSE36994 | 23041383 |
| RNAseq | Erythroid Differentiation | Human, Mouse | GSE53983 | 24637361 |
