# Supplementary material for: GATA1 and PU.1 Bind to Ribosomal Protein Genes in Erythroid Cells: Implications for Ribosomopathies
Source: PLoS One. 2015 Oct 8;10(10):e0140077. doi: 10.1371/journal.pone.0140077 (PMC4598024; doi:10.1371/journal.pone.0140077)
Supplement: S5 Table — Indicated by asterisk are the RP genes that have been found mutated in DBA. (DOC) [file pone.0140077.s009.doc]

**S5 Table**

| Gene Name | Expression (log2(FC)) | GATA1_Ter119neg | GATA1_Ter119pos | PU1_EsEP |
| --- | --- | --- | --- | --- |
| Rpl10a | -1,04 | 2,76 | 2,65 | 0,00 |
| Rpl11* | -0,57 | 0,73 | 0,00 | 0,17 |
| Rpl12 | -1,59 | 1,00 | 0,00 | 0,41 |
| Rpl13 | -1,29 | 0,67 | 0,00 | 0,14 |
| Rpl13a | -1,32 | 5,54 | 1,44 | 0,41 |
| Rpl14 | -1,5 | 3,59 | 2,44 | 0,59 |
| Rpl15* | -2,12 | 0,29 | 0,00 | 0,00 |
| Rpl17 | -0,42 | 0,59 | 0,00 | 1,54 |
| Rpl18 | -1,38 | 2,82 | 0,00 | 2,30 |
| Rpl18a | -1,52 | 1,88 | 0,00 | 1,65 |
| Rpl19 | -1,34 | 1,46 | 1,47 | 0,00 |
| Rpl21 | -0,71 | 1,22 | 0,00 | 0,00 |
| Rpl22 | -1,32 | 0,59 | 0,06 | 0,33 |
| Rpl23 | -1,49 | 0,56 | 0,11 | 0,00 |
| Rpl23a | -9,97 | 4,07 | 2,18 | 0,38 |
| Rpl26* | -1,23 | 1,89 | 0,00 | 0,24 |
| Rpl27a | -1,6 | 1,43 | 0,06 | 0,24 |
| Rpl29 | -0,96 | 1,31 | 0,00 | 0,71 |
| Rpl3 | -1,25 | 3,80 | 1,17 | 0,00 |
| Rpl30 | -1,58 | 0,11 | 0,00 | 0,00 |
| Rpl31 | -2,05 | 1,31 | 0,00 | 0,27 |
| Rpl32 | -1,44 | 2,21 | 0,39 | 0,00 |
| Rpl34 | -1,31 | 0,82 | 0,00 | 0,50 |
| Rpl35 | -1,37 | 2,69 | 0,73 | 2,53 |
| Rpl35a* | -1,21 | 0,77 | 0,00 | 3,17 |
| Rpl36 | -0,67 | 1,82 | 0,06 | 0,00 |
| Rpl36al | -1,46 | 0,87 | 0,00 | 1,64 |
| Rpl37 | -1,12 | 0,89 | 0,45 | 0,00 |
| Rpl37a | -1,55 | 0,37 | 0,00 | 0,00 |
| Rpl38 | -2,18 | 1,38 | 0,00 | 0,06 |
| Rpl39 | -1,09 | 0,65 | 0,00 | 0,00 |
| Rpl39l | -1,34 | 0,00 | 0,00 | 0,00 |
| Rpl3l | -0,6 | 2,26 | 0,70 | 0,00 |
| Rpl4 | -1,33 | 1,42 | 2,96 | 0,00 |
| Rpl41 | -0,54 | 0,00 | 0,00 | 0,00 |
| Rpl6 | -1,16 | 1,69 | 0,17 | 0,00 |
| Rpl7 | -1,17 | 0,79 | 0,00 | 1,53 |
| Rpl7a | -1,06 | 2,52 | 0,56 | 0,12 |
| Rpl8 | -1,1 | 1,02 | 1,32 | 0,92 |
| Rpl9 | -0,74 | 0,72 | 0,00 | 0,00 |
| Rplp0 | -0,83 | 1,38 | 0,49 | 0,35 |
| Rplp1 | -1,1 | 3,15 | 0,06 | 0,63 |
| Rplp2 | -1,05 | 1,71 | 0,00 | 1,04 |
| Rps10* | -1,37 | 0,97 | 0,00 | 0,72 |
| Rps11 | -1,44 | 2,10 | 0,22 | 0,41 |
| Rps12 | -9,97 | 1,10 | 0,31 | 1,15 |
| Rps13 | 0,43 | 0,00 | 0,00 | 0,00 |
| Rps14 | -1,14 | 0,95 | 0,00 | 0,12 |
| Rps15 | -1,12 | 1,01 | 0,00 | 0,17 |
| Rps15a | -2,21 | 0,64 | 0,00 | 0,60 |
| Rps16 | -0,98 | 0,60 | 0,00 | 0,64 |
| Rps17* | -1,9 | 1,03 | 0,00 | 1,40 |
| Rps18 | -1,28 | 1,12 | 0,00 | 0,00 |
| Rps19* | -0,95 | 2,16 | 0,17 | 0,26 |
| Rps2 | -0,65 | 1,98 | 0,11 | 0,00 |
| Rps20 | -1,21 | 0,58 | 0,00 | 1,41 |
| Rps21 | -1,09 | 1,02 | 0,00 | 0,65 |
| Rps23 | -0,67 | 0,97 | 0,00 | 0,00 |
| Rps24* | -1,63 | 0,55 | 0,00 | 0,25 |
| Rps25 | -0,61 | 1,20 | 0,00 | 2,53 |
| Rps26* | -1,19 | 2,04 | 0,51 | 0,00 |
| Rps27* | -2,96 | 0,01 | 0,00 | 0,00 |
| Rps27a | -1,64 | 0,62 | 0,00 | 0,00 |
| Rps27l | -1,4 | 0,58 | 0,00 | 0,00 |
| Rps29* | -0,9 | 1,41 | 0,00 | 0,00 |
| Rps3 | -1,15 | 1,21 | 0,53 | 0,12 |
| Rps3a | -0,86 | 1,19 | 0,00 | 0,71 |
| Rps4x | -1,2 | 0,86 | 0,00 | 0,00 |
| Rps4y2 | -1,62 | 0,02 | 0,00 | 0,00 |
| Rps5 | -1,11 | 0,83 | 0,00 | 0,00 |
| Rps6 | -0,79 | 0,94 | 0,00 | 1,09 |
| Rps7* | -0,84 | 0,38 | 0,00 | 0,00 |
| Rps8 | -1,36 | 0,44 | 0,00 | 4,03 |
| Rps9 | -1,19 | 2,26 | 0,85 | 1,07 |
| Rpsa | -1,36 | 0,38 | 0,00 | 1,23 |
| Fau | -1,27 | 2,54 | 1,07 | 2,57 |
|  |  |  |  |  |
| Gene Name | Expression (log2(FC)) | GATA1_Ter119neg | GATA1_Ter119pos | PU1_EsEP |
| Hbb-b1 | 4,1 | 3,90 | 4,85 | 0,36 |
| Gypa | 3,21 | 7,57 | 12,79 | 0,00 |
| Slc4a1 | 3,84 | 2,99 | 8,13 | 0,00 |
| Nfe2 | 1,75 | 9,35 | 9,30 | 0,91 |
| Tal1 | 1,17 | 5,34 | 5,44 | 0,49 |
| Epor | 0,48 | 5,25 | 5,35 | 0,00 |
| Gata1 | 0,43 | 10,41 | 16,60 | 0,00 |
| Kit | -3,45 | 4,69 | 0,49 | 0,06 |
| Sfpi1 | -3,24 | 3,02 | 1,30 | 0,00 |
| Gata2 | -3,52 | 5,86 | 3,31 | 0,17 |
|  |  |  |  |  |
